# A Rare Case of Partial Anomalous Pulmonary Venous Connection

**DOI:** 10.7759/cureus.50913

**Published:** 2023-12-21

**Authors:** Taishi Fujii, Kai Machida, Sakamoto Daisuke, Nagayoshi Yasuhiro, Tamaki Takano

**Affiliations:** 1 Department of Cardiovascular Surgery, Kanazawa Medical University, Uchinada, JPN; 2 Department of Cardiovascular Surgery, Kanazawa Medical University Hospital, Uchinada, JPN

**Keywords:** mri, mdct, adult congenital heart disease, asd, cut back method, papvc

## Abstract

Partial anomalous pulmonary venous connection (PAPVC), in which the right and left lower pulmonary veins drain into the coronary sinus (CS), is very rare, and only one case has been reported previously. The diagnosis of PAPVC is difficult, as the symptoms may be not specific. Multidetector computed tomography (MDCT) angiography and MRI help in the diagnosis of congenital cardiac anomalies. Our case, who underwent closure of atrial septal defect (ASD) 31 years prior, presented with chest tightness and palpitation. MDCT angiography revealed PAPVC with both lower pulmonary veins draining into the CS. We performed surgical repair of PAPVC by the cut-back method. The postoperative course was uneventful.

## Introduction

In partial anomalous pulmonary venous connection (PAPVC), one or more pulmonary veins return to the right atrium, coronary sinus (CS), and systemic veins. It is a relatively rare condition and is reported as 0.4-0.7% of congenital cardiac anomalies [1]. Rarely, in PAPVC, the right pulmonary veins drain anomalously into the azygos vein or CS, and the left pulmonary veins drain anomalously into CS, a right-sided superior vena cava, or the right atrium. We herein report a very rare case of PAPVC in which bilateral lower pulmonary veins drain into the CS. We discuss the preoperative diagnosis and the surgical method of this unusual case in adult congenital heart disease.

## Case presentation

A 45-year-old Asian woman was admitted to our hospital for complaining of frequent chest tightness and palpitation. She did not have difficulty breathing or a heart murmur and she was not also medicated. She underwent closure of the atrial septal defect (ASD) when she was 14 years old. Her medical history indicated no other congenital diseases. Transthoracic echocardiography (TTE) showed pulmonary hypertension but did not detect other abnormal findings. Multidetector computed tomography (MDCT) angiography revealed PAPVC with right and left lower pulmonary veins draining into an enlarged CS and right and left upper pulmonary veins draining into the left atrium (Video 1).

**Video 1:** MDCT showed both lower PVs draining into the CS and both upper PVs draining into the left atrium MDCT: multidetector computed tomography, CS: coronary sinus, PV: pulmonary vein

**Figure 1:**
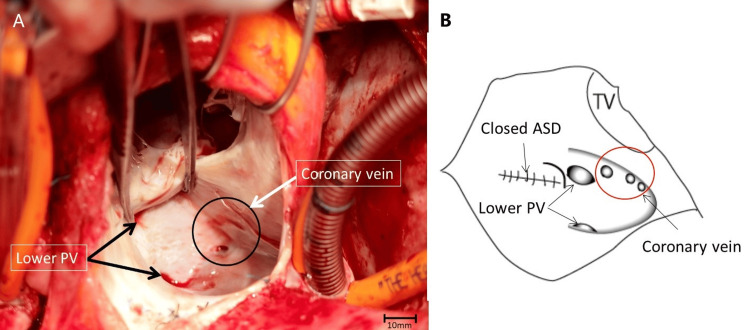
Bilateral lower pulmonary veins opened into the CS cranially from three coronary veins ovale Figure 1B created by the authors PV: pulmonary vein, ASD: atrial septal defect, TV: tricuspid valve, CS: coronary sinus

Transesophageal echocardiography (TEE) showed an L-R shunt but could not reveal the position of the L-R shunt. Magnetic resonance imaging (MRI) presented PAPVC with right and left lower pulmonary veins draining into the CS. The pulmonary-to-systemic flow ratio was 2.14 and the L-R shunt ratio was 53% in cardiac catheterization. She was diagnosed with PAPVC, which was not noticed at the initial surgery, and a re-operation was performed. Cardiopulmonary bypass was established with bicaval and ascending aorta cannulation after median sternotomy. Cardiac arrest was achieved with antegrade cold crystalloid cardioplegia. Through a right atriotomy, we found bilateral lower pulmonary veins opened into the CS cranially from three coronary veins ovale (Figure 1).

ASD was directly closed with a suture in the previous operation. We incised the intra-atrial septum, and re-routing was performed with the autologous pericardium. The pericardium was sutured between the pulmonary and coronary veins to ensure that the pulmonary veins drain into the left atrium, whereas the CS drains into the right atrium. The postoperative course was smooth. The patient was discharged with no morbidity. TTE showed no L-R shunt, and the pulmonary-to-systemic flow ratio was 1.0. MDCT angiography showed bilateral lower pulmonary veins drained into the left atrium and the CS drained into the right atrium (Video 2).

**Video 2:** Bilateral lower pulmonary veins drained into the left atrium

The patient was discharged on postoperative day 13 and has had no shunt during a five-year follow-up.

## Discussion

PAPVC is a congenital developmental disorder in which abnormal connections from one to three out of four pulmonary veins drain into the right atrium, coronary sinus, venae cavae, or their tributaries [2]. The incidence of PAPVC is 0.4-0.7% of all congenital heart disease in autopsy cases. PAPVC draining into the CS is rare, at 0.09-2.2 % of PAPVC [1,3]. The anomalous pulmonary venous drainage is ten times as common from the right lung as from the left lung. Partial anomalous drainage from both lungs is 7.0-9.0% of PAPVC [1,2]. In previous reports, in three cases [4], both right and left lower pulmonary veins drained into the CS, and in only one case, both right and left lower pulmonary veins drained into the CS [5]. Our case showed both right and left lower pulmonary veins draining into the CS, which is considered very rare.

The diagnosis of PAPVC has been difficult because symptoms that may present in PAPVC are not specific. Major symptoms include dyspnea, palpitation, edema, and fatigue [6]. PAPVC is sometimes misdiagnosed as primary pulmonary hypertension or was diagnosed incidentally [6] because of this specificity. TTE remains the first choice of non-invasive imaging modality to diagnose PAPVC although its sensitivity is relatively low at 67% [6]. In our case, PAPVC was not documented at the first operation and had not been diagnosed during follow-up after the first surgery. TEE is considered more sensitive than TTE, but we could not obtain sufficient information because of the limited acoustic window in the presenting case. MDCT angiography and MRI have gained progressive importance in the non-invasive evaluation of congenital cardiac anomalies [7-10]. In our case, the diagnosis and the anatomical features as the presence and number of anomalous veins were obtained by MDCT angiography and MRI.

Surgical correction of PAPVC into CS is by the cut-back method and the left atrial wall flap technique [11-13]. The cut-back method is rerouting the pulmonary veins into the left atrium and the coronary veins into the right atrium with a patch after cutting back a roof of the coronary sinus opening to the left atrium. We chose the cut-back method because we easily found orifices of pulmonary veins in the coronary sinus. There was enough space to make a switch between the pulmonary and coronary veins in the presenting case. We could accomplish the repair without pulmonary venous and coronary vein obstruction. No conduction problem was observed in the ECG, and no residual shunt and no pulmonary venous obstruction were not shown with TTE and MDCT angiography postoperatively although long-time follow-up is imperative.

## Conclusions

In conclusion, we experienced a very rare case of PAPVC with both lower pulmonary veins draining into CS. We could diagnose PAPVC by MDCT angiography. We performed surgery with the cut-back method and gained favorable results. The patient has had no obstruction of blood flow from pulmonary veins into CS and no residual shunt for more than five years of follow-up. It is important to recognize the various types of PAPVC because the diagnosis may be incorrect before appearing in MDCT angiography and MRI in adult congenital heart disease.
